# Expression patterns of platinum resistance-related genes in lung adenocarcinoma and related clinical value models

**DOI:** 10.3389/fgene.2022.993322

**Published:** 2022-11-24

**Authors:** Zhe Wang, Lin Mu, He Feng, Jialin Yao, Qin Wang, Wenxiao Yang, Huiling Zhou, Qinglin Li, Ling Xu

**Affiliations:** ^1^ Department of Oncology, Yueyang Hospital of Integrated Traditional Chinese and Western Medicine, Shanghai University of Traditional Chinese Medicine, Shanghai, China; ^2^ Department of Ophthalmology, Longhua Hospital Shanghai University of Traditional Chinese Medicine, Shanghai, China; ^3^ Cancer Hospital of the University of Chinese Academy of Sciences (Zhejiang Cancer Hospital), Zhejiang, China

**Keywords:** lung adenocarcinoma, platinum resistance, immune cells, M6A, bioinformatics, TCGA

## Abstract

The purpose of this study was to explore platinum resistance-related biomarkers and mechanisms in lung adenocarcinoma. Through the analysis of gene expression data of lung adenocarcinoma patients and normal patients from The Cancer Genome Atlas, Gene Expression Omnibus database, and A database of genes related to platinum resistance, platinum resistance genes in lung adenocarcinoma and platinum resistance-related differentially expressed genes were obtained. After screening by a statistical significance threshold, a total of 252 genes were defined as platinum resistance genes with significant differential expression, of which 161 were up-regulated and 91 were down-regulated. The enrichment results of up-regulated gene Gene Ontology (GO) showed that TOP3 entries related to biological processes (BP) were double-strand break repair, DNA recombination, DNA replication, the down-regulated gene GO enriches the TOP3 items about biological processes (BP) as a response to lipopolysaccharide, muscle cell proliferation, response to molecule of bacterial origin. Gene Set Enrichment Analysis showed that the top three were e2f targets, g2m checkpoint, and rgf beta signaling. A prognostic model based on non-negative matrix factorization classification showed the characteristics of high- and low-risk groups. The prognostic model established by least absolute shrinkage and selection operator regression and risk factor analysis showed that genes such as HOXB7, NT5E, and KRT18 were positively correlated with risk score. By analyzing the differences in m6A regulatory factors between high- and low-risk groups, it was found that FTO, GPM6A, METTL3, and YTHDC2 were higher in the low-risk group, while HNRNPA2B1, HNRNPC, TGF2BP1, IGF2BP2, IGF2BP3, and RBM15B were higher in the high-risk group. Immune infiltration and drug sensitivity analysis also showed the gene characteristics of the platinum-resistant population in lung adenocarcinoma. ceRNA analysis showed that has-miR-374a-5p and RP6-24A23.7 were lower in the tumor expression group, and that the survival of the low expression group was worse than that of the high expression group. In conclusion, the results of this study show that platinum resistance-related differentially expressed genes in lung adenocarcinoma are mainly concentrated in biological processes such as DNA recombination and response to lipopolysaccharide. The validation set proved that the high-risk group of our prognostic model had poor survival. M6A regulatory factor analysis, immune infiltration, and drug sensitivity analysis all showed differences between high and low-risk groups. ceRNA analysis showed that has-miR-374a-5p and RP6-24A23.7 could be protective factors. Further exploration of the potential impact of these genes on the risk and prognosis of drug-resistant patients with lung adenocarcinoma would provide theoretical support for future research.

## 1 Introduction

Globally, the mortality rate of lung cancer is the highest among all tumors (Xia et al., 2022). Non-small cell lung cancer (NSCLC), which accounts for 80% of all lung cancer cases (Miller et al., 2016), can be divided into three main pathological subtypes: adenocarcinoma (40%), squamous cell carcinoma (30%), and large cell carcinoma (15%) (Ruiz-Cordero and Devine, 2020; Travis et al., 2016). The standard first-line treatment is still platinum-based combined chemotherapy (Scagliotti et al., 2002; Schiller et al., 2002). Although chemotherapy can bring benefits to lung adenocarcinoma (LUAD) patients, the median progression-free survival time is only 5.5 months (West et al., 2019), and drug resistance is inevitable. Although many studies have explored the mechanism of platinum drug resistance, there is still no clear mechanism or targets of platinum drug resistance, and few research results can be used in clinical application. Therefore, we aimed to explore the potential biomarkers and mechanisms of platinum-based drug resistance genes in LUAD, establish a prognostic model, and conduct related research on clinical prognosis and risk.

The rapid development of immunotherapy over the last decade has led to the improvement of immune checkpoint inhibitors, which has improved the clinical outcomes of some patients with advanced cancer and changed the treatment status of NSCLC (Osmani et al., 2018; Queirolo and Spagnolo, 2017). Therefore, attention has been paid to immune cell infiltration, the role of immune infiltration in the occurrence, development, and prognosis of platinum-based LUAD, prognostic information, and predicting the efficacy of immunotherapy. Drug sensitivity analysis can also provide guidance for follow-up treatment of platinum-resistant patients with LUAD. Several studies have shown that mRNA methylation plays an important role in the occurrence and development of some cancers (e.g., glioblastoma, renal clear cell carcinoma, and pancreatic cancer) (Cui et al., 2017; Du et al., 2020; Geng et al., 2020; Lan et al., 2019; Wang et al., 2020). These studies indicated that the development of tumors may be related to the expression of key genes related to the function of the m6A regulator. However, there is no research on the m6A methylation regulatory factor with respect to platinum drug resistance in LUAD.

**FIGURE 1:**
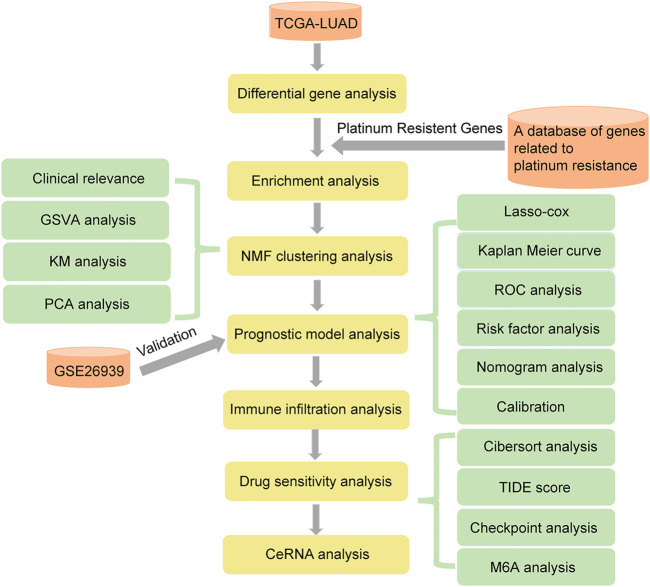
Workflow (Modify database name).

In this study, we aimed to identify the characteristics of platinum drug resistance genes in LUAD, and explore the characteristics of patients after drug resistance, to pave the way for further study of drug resistance mechanisms. We first explored the expression patterns of platinum-resistant genes related to LUAD in The Cancer Genome Atlas (TCGA), Gene Expression Omnibus (GEO), and A database of genes related to platinum resistance. Functional annotations and channel analysis of different platinum resistance genes were performed through Gene Ontology (GO), Kyoto Encyclopedia of Genes and Genomes (KEGG) and Gene Set Enrichment Analysis (GSEA). Subsequently, we evaluated the ability to transform into clinical applications through non-negative matrix factorization (NMF) cluster analysis and the establishment of a prognosis model. Immune infiltration and drug sensitivity analyses were used to evaluate the possible applicability of patients with LUAD platinum resistance to other clinical treatments. The results of this study provide guidance for the development of clinical drugs for platinum-resistant patients with LUAD. Workflow is shown in Figure 1.

## 2 Materials and methods

### 2.1 Expression datasets and bioinformatic analysis

#### 2.1.1 Data and preprocessing

We downloaded the gene expression profile, miRNA expression profile, and clinical information data of LUAD from the TCGA database (n = 585) (https://tcga-data.nci.nih.gov/tcga/). A total of 912 genes related to platinum resistance were downloaded from A database of genes related to platinum resistance (http://ptrc-ddr.cptac-data-view.org). Finally, expression data and clinical information of 116 LUAD samples (data number GSE26939) were downloaded from the GEO database (https://www.ncbi.nlm.nih.gov/geo/query/acc.cgi?acc=GSE26939) for use as the verification dataset.

#### 2.1.2 Differentially expressed gene (DEG) analysis

**FIGURE 2:**
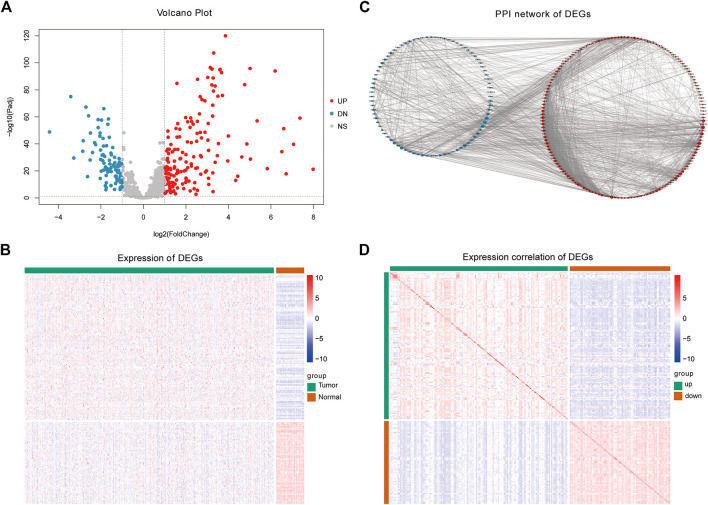
Differential expression analysis results. **(A)** Difference analysis volcano plot. **(B)** Heatmap of differentially expressed platinum resistance genes. **(C)** Differentially expressed platinum resistance gene PPI network. **(D)** Heatmap of correlations of differentially expressed platinum resistance genes.

We analyzed the differences in gene expression between cancer and normal patients using the R package DESeq2 (v1. 32.0) (Love et al., 2014). We set | log2fold change | (| log2fc |) ≥ 1 and adjust *p* value <0.05 as the threshold of differential genes; log2fc ≥ 1 and adjust *p* value <0.05 gene was used to identify upregulated genes, while log2fc ≤ −1 and adjust *p* value ≤0.05 were used to identify downregulated genes. Cisplatin-based chemotherapy is a common method to treat LUAD. However, after developing resistance to cis-diammine di chloroplatinum (CDDP), a considerable number of patients’ tumors recurred. Therefore, screening patients with primary resistance to cisplatin in the LUAD population can maximize the clinical benefit. To further reveal the biological functions affected by the DEGs related to platinum resistance, the intersection of platinum resistance genes and DEGs was defined as PRR-DEGs. We used a volcano map and heatmap to visualize platinum resistance genes with significant differential expression. The volcano map was drawn using the R package ggplot2 (v3.3.5), the heat diagram was drawn using the R package pheatmap (v1.0.12) (Figure 2A). The detailed information is shown in Supplementary Table S1.

The expression of different genes is interrelated, especially among genes that regulate the same biological process. Therefore, to reveal the relationships between the PRR-DEGs, a protein–protein interaction network (PPI) was constructed based on the platinum resistance-DEGs. Using the String database (https://www.string-db.org) (Mering, 2003), the above genes were used as input, and the default confidence threshold was 0.4 (Figure 2B). The PPI network was constructed, and the visualization was carried out using the Cytoscape (v3.8.2) (Shannon et al., 2003) software. The expression correlation of PRR-DEGs was calculated and visualized using a nomogram.

#### 2.1.3 Functional enrichment analysis

**FIGURE 3:**
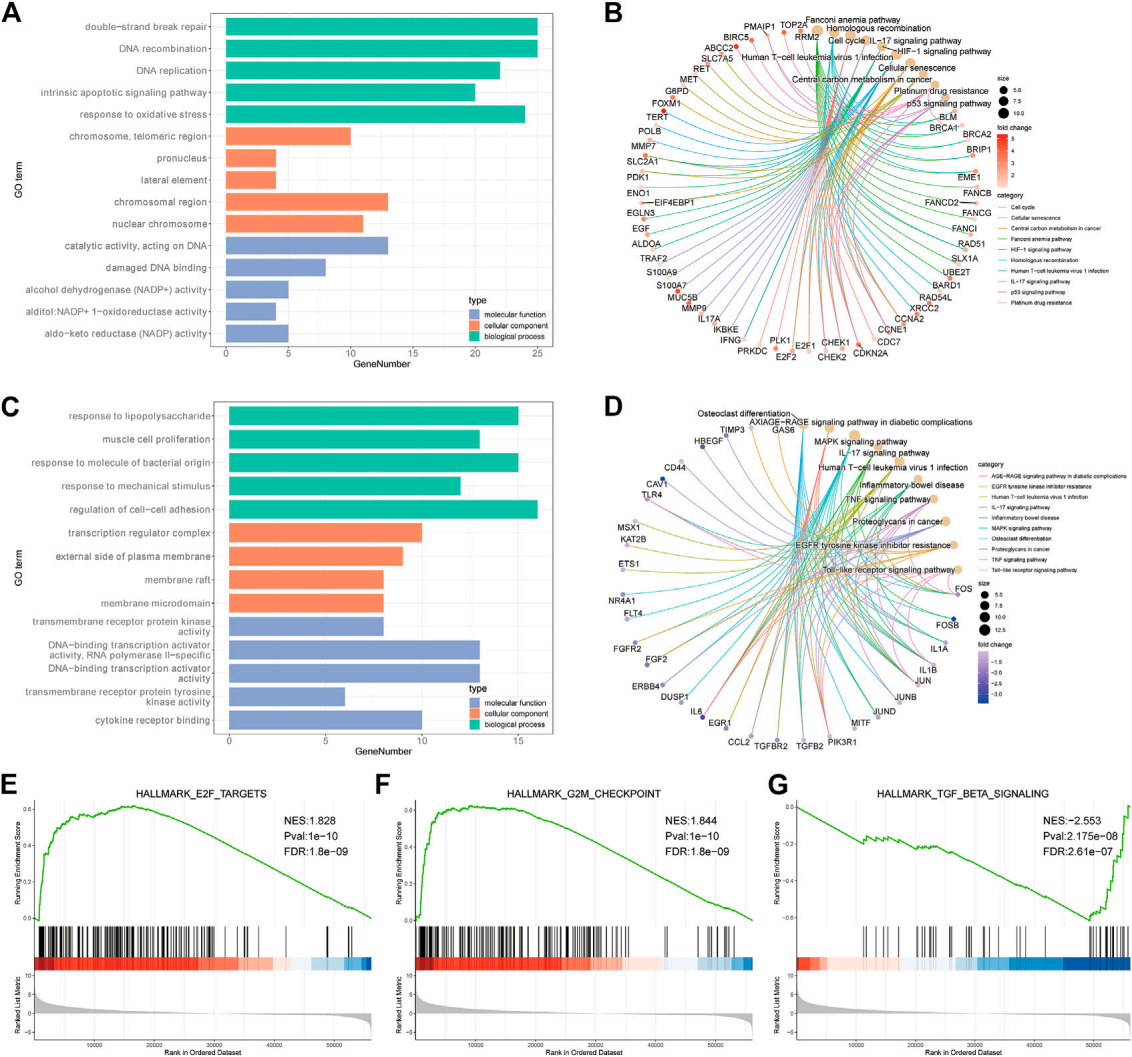
Enrichment results of GO, KEGG and GSEA. **(A)** GO enrichment analysis bar chart of up-regulated genes (TOP5 of BP, CC, and MF). **(B)** up-regulating the enrichment result of gene KEGG. **(C)** GO enrichment analysis bar chart of down-regulated genes (TOP5 of BP, CC and MF). **(D)** down-regulating the enrichment result of gene KEGG. **(E–G)**: GSEA analysis of TOP3 results.

GO (Http://geneontology.org/) is a common method for large-scale functional enrichment of genes in different dimensions and levels. It is generally carried out from three levels: biological process (BP), molecular function (MF), and cellular component (CC) (Harris et al., 2004). The R package cluster Profiler (v4.0.5) (Wu et al., 2021) was used for GO functional annotation analysis of all the genes with significantly different expression levels to identify the biological processes and pathways with significant enrichment. The enrichment results were visualized using the R package GOplot (v1.0.2) (Walter et al., 2015). The significance threshold of enrichment analysis was set as adjust *p* value ≤0.05 (Figures 3A,C).

KEGG (https://www.kegg.jp/) is a utility database resource for understanding advanced functions and biological systems (such as cells, organisms and ecosystems), genome sequencing and other high-throughput experimental techniques generated from molecular level information, especially large molecular data sets. It was established in 1995 by Kanehisa Laboratory of Bioinformatics Center of Kyoto University, Japan. The significance threshold of enrichment analysis was set as p adjust *p* value ≤0.05 (Figures 3B,D).

GSEA is a calculation method used to determine whether a group of predefined genes shows a statistical difference between two biological states. It is usually used to estimate changes in pathways and biological process activities in expression dataset samples (Subramanian et al., 2005). To study the differences in biological processes between the two groups of patients, we used the gene expression profile dataset from the MSigDB database (Liberzon et al., 2015) (https://www.gsea-msigdb.org) (Figures 3E–G). The detailed information is shown in Supplementary Table S2.

#### 2.1.4 NMF cluster analysis

**FIGURE 4:**
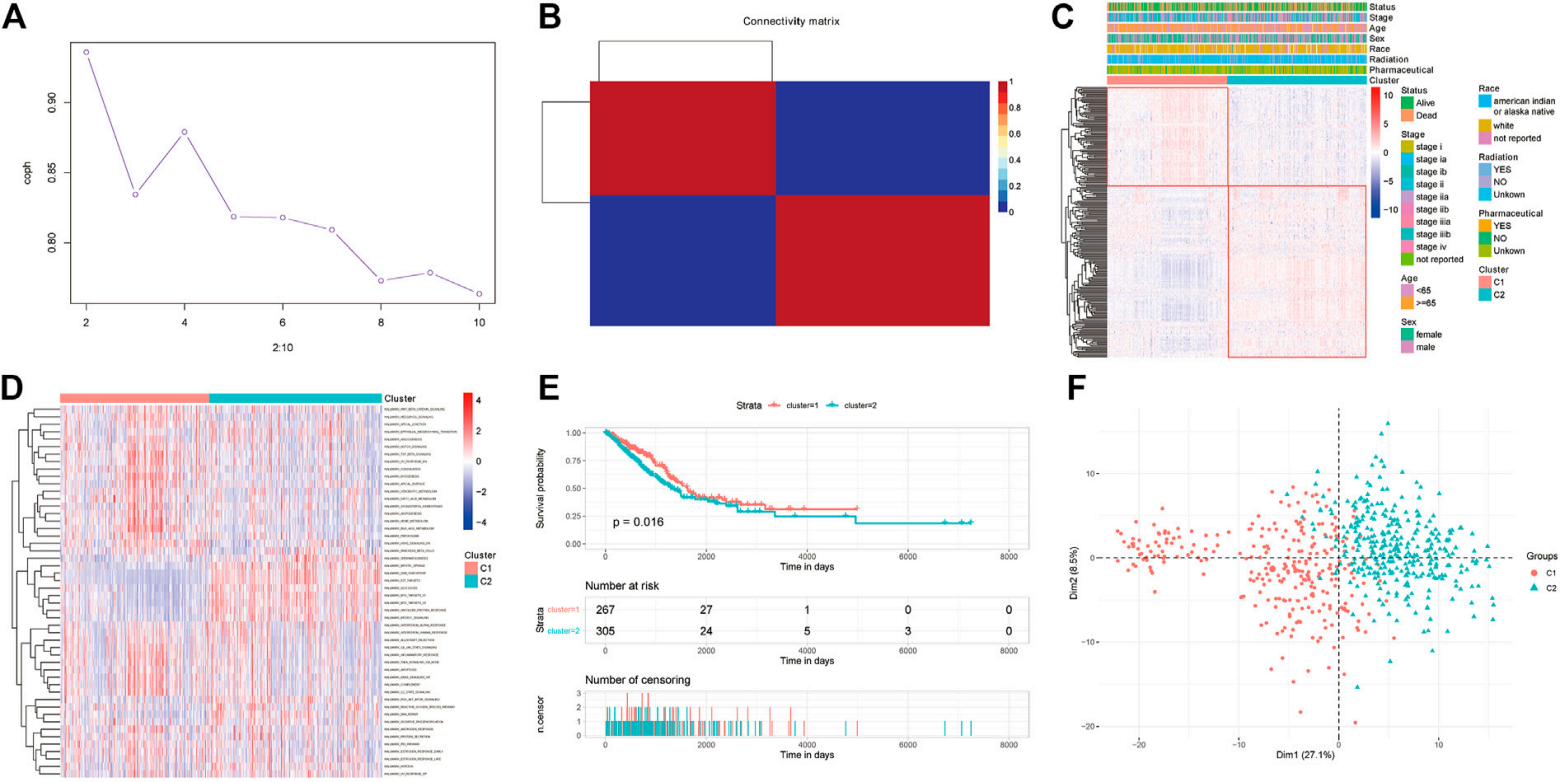
NMF clustering results. **(A)** NMF cluster cophenetic *versus* number of clusters. **(B)** NMF clustering results. **(C)** Heatmap of differentially expressed platinum resistance genes associated with clinical information. **(D)** Heatmap of GSVA analysis of cancer hallmarks. **(E)** Survival differences for NMF clustering. **(F)** Plot of PCA results for NMF clustering categories.

Nonnegative matrix factorization, referred to as NMF, is a matrix factorization method proposed by Lee and Seung in the journal Nature in 1999 (Lee and Seung, 1999). It makes all the decomposed components non-negative (requiring purely additive description), and at the same time realizes nonlinear dimension reduction. The samples were analyzed by NMF unsupervised cluster analysis, which was realized using the NMF package in R (v0.23.0) (Zushi, 2021). The correlation between the expression of PRR-DEGs and clinical features (including race, stage, age, and sex) based on NMF classification was visualized (Figures 4A–C). Gene set variation analysis, referred to as GSVA, is a non-parametric and unsupervised algorithm. Unlike GSEA, GSVA does not need to group samples in advance and can calculate the enrichment scores of specific gene sets in each sample. In other words, GSVA transforms gene expression data from a single gene as a feature expression matrix to a specific gene set as a feature expression matrix. To further analyze differences between NMF classifications, GSVA analysis was implemented using the GSVA package (v1.40.1). Finally, the features of NMF classification were visualized using the FactoMineR (v2.4) and factoextra packages (v1.0.7) (Figures 4D–F).

#### 2.1.5 Prognostic model construction

**FIGURE 5:**
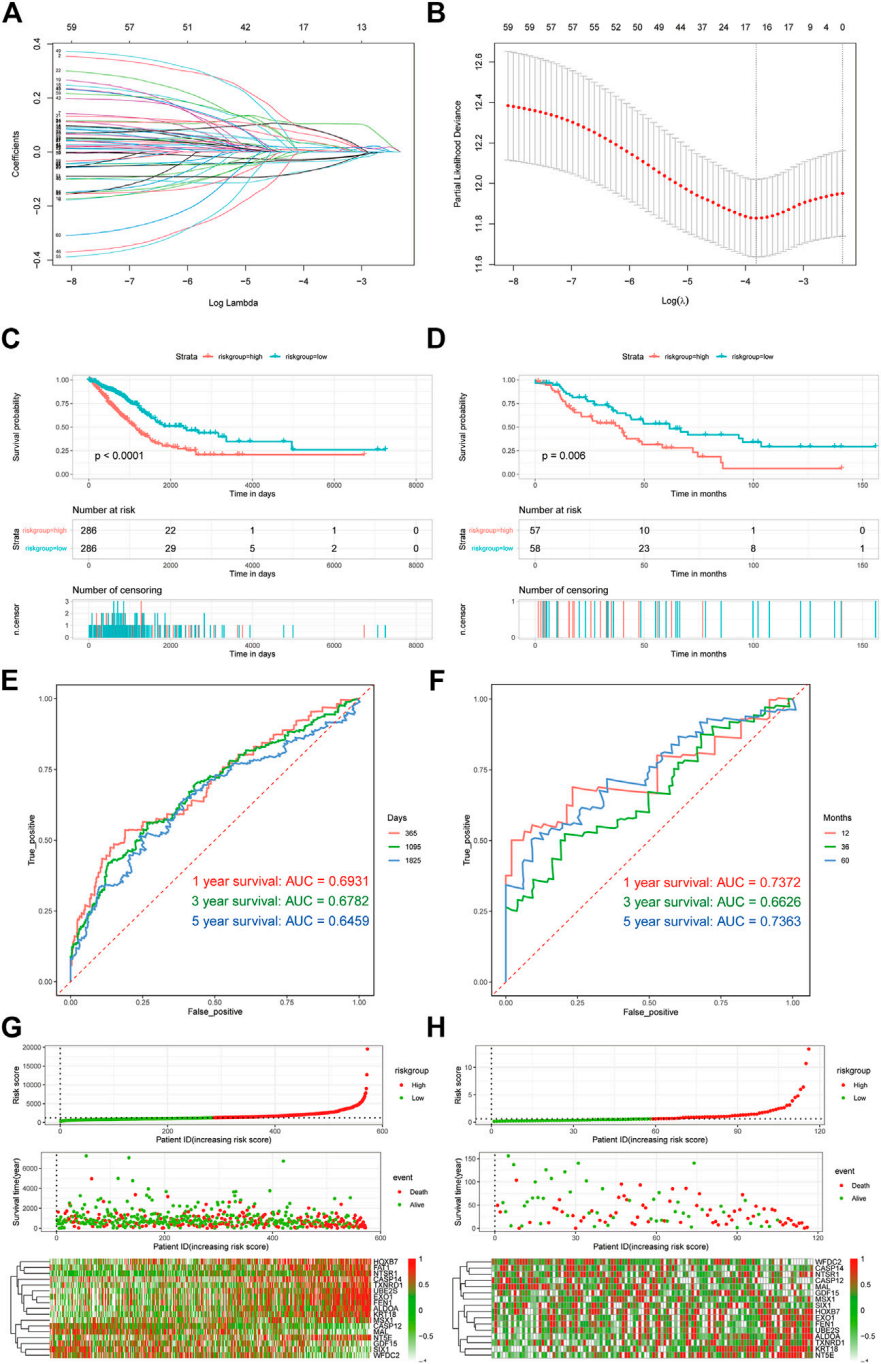
Construction and evaluation of prognostic models. **(A,B)** Lasso-cox regression analysis graph. **(C,D)** Survival analysis of TCGA LUAD and GSE16939. **(E,F)** Time ROC curve analysis of TCGA LUAD and GSE16939. **(G,H)** Association factor analysis of TCGA LUAD and GSE16939.

Owing to the importance of platinum resistance in the treatment of LUAD, different patients may have different platinum resistance states; as such, it is extremely feasible to construct a diagnostic model based on differentially expressed platinum resistance genes. Here, firstly, we used the least absolute shrinkage and selection operator (LASSO) regression method to screen differentially expressed platinum resistance genes; the R package glmnet (v4.1–2) was used to realize this method and select the best lambda symbol value. After regression, only genes with coefficients other than 0 were retained (Figures 5A,B).

To analyze the relationship between the prognosis model and survival status, we used Kaplan–Meier survival analysis and risk factor analysis. Then, to verify the predictive efficiency of the diagnostic model, receiver operating characteristic (ROC) curves were drawn using the R package pROC (v1.18.0) (Robin et al., 2011). The area under the curve (AUC) of 1 year, 3 years, and 5 years were evaluated. To further prove the robustness of the model prediction, external data (GSE26939) were used for verification (Figures 5C–F).

To further verify the efficacy of the prognostic model of PRR-DEGs, we incorporated clinical indicators into the model, evaluated the univariate and multifactorial prognostic models of clinical factors using the survival package (v3.2.11), and displayed them as forest maps. In addition, we took clinical factors into account and used the rms package (v6.2-0) to construct clinical prediction nomogram and corresponding calibrate correction charts (Figures 5G,H).

**FIGURE 6:**
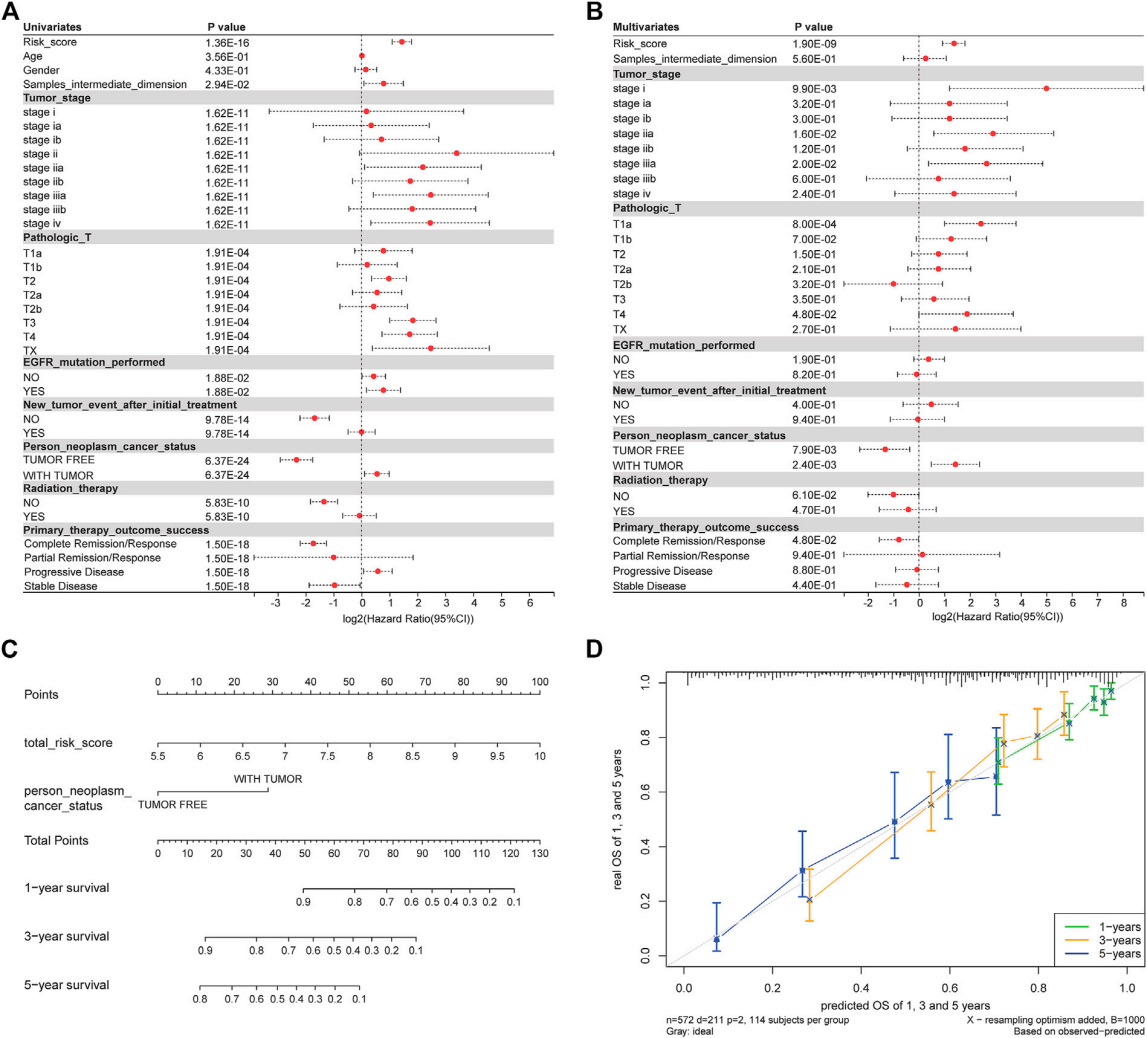
Clinical factors are associated with prognosis. **(A)** Univariate regression analysis results. **(B)** Results of multivariate regression analysis. **(C)** Nomogram analysis. **(D)** Calibration correction chart.

We investigated whether clinical features are related to prognosis. Univariate Cox regression analysis showed that risk score, sample intermediate dimension, tumor stage, person neoplasm cancer status were significantly correlated with OS (Figure 6A). Finally, these univariate prognostic variables were used as covariates of multiple cox regression analysis. The results showed that risk score and tumor status were independent prognostic factors related to OS (Figure 6B).

In order to evaluate whether our model can effectively predict the prognosis of patients in the clinical environment, we incorporated OS-related factors into the model and constructed a nomogram (Figure 6C) to predict the OS: 1, 3, and 5-year survival rates of patients. The nomogram model once again confirms the reliability and prospective clinical applicability of the risk model. At the same time, we Calibration the nomogram and found that the predicted results were highly correlated with the actual survival rate (Figure 6D).

#### 2.1.6 Immune infiltration analysis

The immune microenvironment mainly consists of immune cells, inflammatory cells, fibroblasts, interstitial tissues, and various cytokines and chemokines, and is a loaded comprehensive system. The infiltration analysis of immune cells in tissues plays an important guiding role in disease research and treatment prognosis prediction.

**FIGURE 7:**
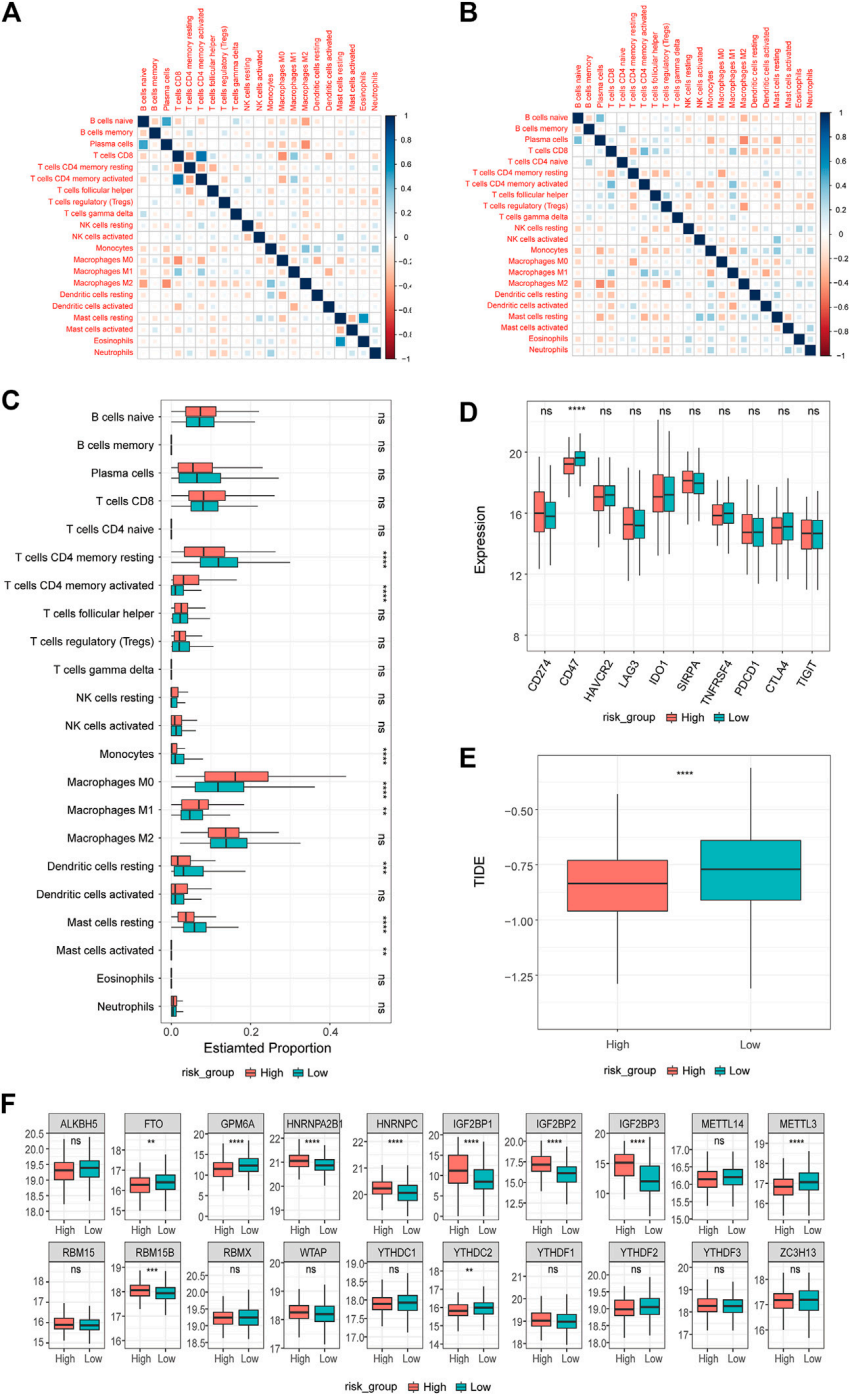
Results of immune infiltration analysis. **(A,B)** Correlation of immune infiltration in high-risk and low-risk groups. **(C)** Differences in immune infiltration between high and low risk groups. **(D)** Differences in immune checkpoints between high and low risk groups. Differences in **(E)** TIDE between high and low risk groups. Differences of **(F)** m6A regulon in high and low risk groups.

To further explore the relationship between differentially expressed prognostic platinum resistance genes and the infiltration level of immune cells, CIBERSORT (Steen et al., 2020) was used to evaluate the infiltration level of immune cells. The contents of 22 kinds of immune cells in each patient were calculated based on the LM22 background gene set provided by the CIBERSORT website (https://cibersort.stanford.edu/) to reflect the infiltration level. The results were visualized using box diagrams drawn by the R package ggplot2 (v3.3.5) (Figures 7A–D). Significant differences between high and low immune cell expression groups of patients may be related to the prognosis of PRR-DEGs. We used the R package ggExtra (v0.9) and a *p*-value < 0.01 to identify extremely significant differences in levels of immune cell infiltration, DEGs, and the prognosis of platinum resistance gene expression; the results were visualized using scatter plots and correlation curve fitting. At the same time, we checked the high- and low-risk groups of the immune checkpoints (CD274, CD47, HAVCR2, LAG3, IDO1, SIRPA, TNFRSF4, YTCN1, PDC D1, CTLA4, and TIGIT). The tumor immune dysfunction and exclusion (TIDE) score reflects the sensitivity to immune checkpoints, and the TIDE score calculated for each tumor sample could be used as a substitute biomarker to predict responses to immune checkpoint blocking (Figures 7E,F).

N-methyladenine (m6A) modification is the most common and abundant RNA modification in eukaryotes. To explore differences in m6A between high and low groups, we used m6A regulatory factor data from Yongsheng Li et al. (Li Y et al., 2019), including 11 readers, 7 writers, and 2 erasers.

#### 2.1.7 Drug sensitivity analysis

The LUAD cell line-drug action dataset was obtained from the Genomics of Drug Sensitivity in Cancer database (GDSC www.cancerRxgene.org) (Yang et al., 2012). The drug sensitivity of the expression data in TCGA-LUAD was analyzed using the R package oncoPredict (v0.2) (Maeser et al., 2021), and the sensitivity differences of high- and low-risk groups to different drugs were compared (Figure 9).

#### 2.1.8 Construction of ceRNA network

To analyze the relationship between DEGs and miRNA and lncRNA in the post-transcriptional stage, we collected data from the Tarbase (https://dianalab.e-ce.uth.gr/html/diana/web/index.php?R=tarbasev8) (Karagkouni et al., 2018) and TargetScan (https://www.targetscan.org/vert_72/) (Agarwal et al., 2015) databases for miRNA molecules that interact with PRR-DEGs. Using two databases improved the accuracy, and eulerr (v6.1.1) was used to draw the Wayne diagram. Then, using lncbase predicted v.2 (https://dianalab.e-ce.uth.gr/html/diana/web/index.php?R = lncbasev2/index-predicted) (Paraskevopoulou et al., 2016) and the StarBase V2.0 database (https://starbase.sysu.edu.cn/starbase2/index.php) (Maeser et al., 2021), we found that miRNA and lncRNA molecules can bind to, and then construct, the ceRNA network. In addition, we analyzed the expression differences of miRNA and lncRNA in the normal tumor group and divided the samples into high- and low-expression groups according to the average expression of miRNA and lncRNA. Finally, we performed Kaplan–Meier survival analysis (Figure 10).

#### 2.1.9 Statistical analysis

All data calculations and statistical analyses are carried out in the R language (v4.1.0). For comparison between the two groups, we used a variance test, *p* ≤ 0.05 was considered statistically significant.

## 3 Results

### 3.1 DEG analysis

To reveal biological differences between LUAD patients and healthy people at the transcriptome level, DEG analysis was conducted between the two groups. After screening by a statistical significance threshold, a total of 252 genes were defined as platinum resistance genes with significant differential expression (Figures 2A,B), of which 161 were upregulated and 91 were downregulated. The PPI network diagram (Figure 2C) of PRR-DEGs was constructed using the String database. In addition, we calculated the correlation of differentially expressed platinum resistance genes, and found that the correlation between upregulated and downregulated genes was closer (Figure 2D).

### 3.2 Functional enrichment analysis

In order to further reveal the biological functions and processes affected by the differential expression of platinum resistance genes, up-regulated and down-regulated genes were enriched by GO, KEGG, and GSEA and visualized in various forms. The enrichment results of up-regulated gene GO showed that TOP5 entries related to biological processes (BP) were double-strand break repair, DNA reconstruction, DNA replication, intrinsic apoptotic pathway, and a response to oxidative stress (Figure 3A), KEGG enriched to cell cycle, cellular senescence, IL-17 signaling pathway, p53 signaling pathway, platinum drug resistance *etc.* (Figure 3B). The down-regulated gene GO enriches the TOP5 items about biological processes (BP) as a response to lipopolysaccharide, muscle cell proliferation, response to molecule of bacterial origin, response to mechanical stimulus, and regulation of cell-cell adhesion (Figure 3C). Down-regulated gene KEGG enrichment is mainly related to the MAPK signaling pathway, IL-17 signaling pathway, EGFR tyrosine kinase inhibitor resistance, TNF signaling pathway, and other related pathways (Figure 3D). In addition, GSEA analysis showed that TOP3 was E2F targets, G2M checkpoint, TGFβ signaling pathway (Figures 3E–G).

### 3.3 NMF clustering analysis

We molecular-typed the data of TCGA-LUAD according to the PRR-DEGs. In the NMF method (Figure 4A), the abscissa corresponding to the first sharp decline of the cophenetic graph is the optimal cluster number. The results (Figure 4B) showed that the samples of LUAD could be divided into two categories. Then, we visualized the relationships between the PRR-DEGs and clinical information such as stage, race, age, and sex (Figure 4C), and identified that PRR-DEGs can basically be classified according to NMF. In addition, we calculated the GSVA analysis scores of cancer hallmarks (Figure 4D). WNT beta-catenin signaling and hedgegcg signaling, among others, scored higher in the C1 category, g2 checkpoint and e2f targets, among others, scored higher in the C2 category. The survival analysis of the two classifications of NMF (Figure 4E) showed that the survival rate of C1 was slightly higher than that of C2, and was statistically significant (*p* < 0.05). Principal component analysis (PCA) analysis suggested that most samples of the two categories of NMF can be separated (Figure 4F).

### 3.4 Construction and evaluation of prognosis model

We constructed a prognosis model based on the PRR-DEGs in order to translate the research results into practical clinical application. First, 252 PRR-DEGs were screened using the LASSO regression method (Figures 5A,B). There were 17 genes with non-zero retention coefficients; therefore, we constructed a prognosis model with 17 genes, and the influence coefficient of each gene was the coefficient of the LASSO regression results. To verify the prognostic efficacy of the prognostic model, survival analysis (Figures 5C,D) was carried out based on TCGA-LUAD data and the validation dataset (GSE26939). The survival of the high-risk group was poor. In addition, the predicted ROC curve was drawn and the AUC was calculated. The results show that the prediction model has excellent prediction efficiency for both sets of data, and the AUC was ∼0.7 (Figures 5D–F). Finally, in order to evaluate the correlation trend between each gene and risk score, risk factor analysis (Figures 5G,H) was carried out. Genes such as HOXB7, NT5E, and KRT18 were positively correlated with risk score in the two groups of data.

We investigated whether clinical features are related to prognosis. Univariate Cox regression analysis showed that the prognosis model gene, KRAS mutation, stage, and so on, were significantly correlated with OS (Figure 6A). Finally, these univariate prognostic variables were used as covariates of multivariate Cox regression analysis. The results showed that all of the prognostic model genes except CASP14 were independent prognostic factors related to OS (Figure 6B). The 17 genes are ALDOA, CASP12, CASP14, EXO1, FAT1, FEN1, GDF15, HOXB7, KRT18, MAL, MSX1, NT5E, NTSR1, SIX1, TXNRD1, UBE2S, and WFDC2. The risk score formula is risk score = 0.118495*exp (ALDOA) - 0.003607*exp (CASP12) + 0.01103671*exp (CASP14) - 0.03671*exp (EXO1) + 0.074027*exp (FAT1) + 0.058166*exp (FEN1) - 0.04225*exp (GDF15) + 0.061475*exp (HOXB7) + 0.105,367*exp (KRT18) - 0.10004*exp (MAL) + 0.134,121*exp (MSX1) + 0.099919*exp (NT5E) + 0.014771*exp (NTSR1) - 0.11253*exp (SIX1) + 0.024867*exp (TXNRD1) + 0.042712*exp (UBE2S) - 0.04043*exp (WFDC2).

To evaluate whether our model could effectively predict the prognosis of patients in clinical environments, we incorporated OS-related factors into the model and constructed a Nomogram (Figure 6C) to predict OS 1-, 3-, and 5-year survival rates. The Nomogram model again confirmed the reliability and prospective clinical applicability of the risk model. At the same time, upon calibration to the Nomogram, the predicted results were highly correlated with the actual survival rate (Figure 6D).

### 3.5 Immune infiltration analysis

To further explore the degree of immune cell infiltration in patients, the CIBERSORT method was used to calculate the degree of infiltration in all samples based on 22 kinds of background genes of immune cells. First, the correlation between the infiltration degree of immune cells in high- and low-risk groups (Figures 7A,B) was calculated. In the high- or low-risk group, the correlation between macrophage M, naïve B cells, and plasma cells was high. In addition, we examined the differences in immune cell infiltration for the different groups (Figure 7C) and found significant differences in 8 of the 22 kinds of immune cells in both groups. Resting T cells CD4 memory, resting dendritic cells, resting mast cell, and monocytes have a higher degree of infiltration in the low-risk group, while T cells CD4 memory activated, macrophage M0, macrophage M1, and mast cell activated have a higher degree of infiltration in the high-risk group. In addition, we examined the differential expression of ten common immune checkpoints in the high- and low-risk groups (Figure 7D) and found that only CD47 was highly expressed in low-risk groups. We further explored the differences in TIDE scores between the two groups (Figure 7E) and found that the TIDE score of the low-risk group was higher. Then, we analyzed the differences in m6A regulatory factors between the high- and low-risk groups (Figure 7F)and found that FTO, GPM6A, METTL3, and YTHDC2 were higher in the low-risk group, while HNRNPA2B1, HNRNPC, TGF2BP1, IGF2BP2, IGF2BP3, and RBM15B were higher in the high-risk group.

**FIGURE 8:**
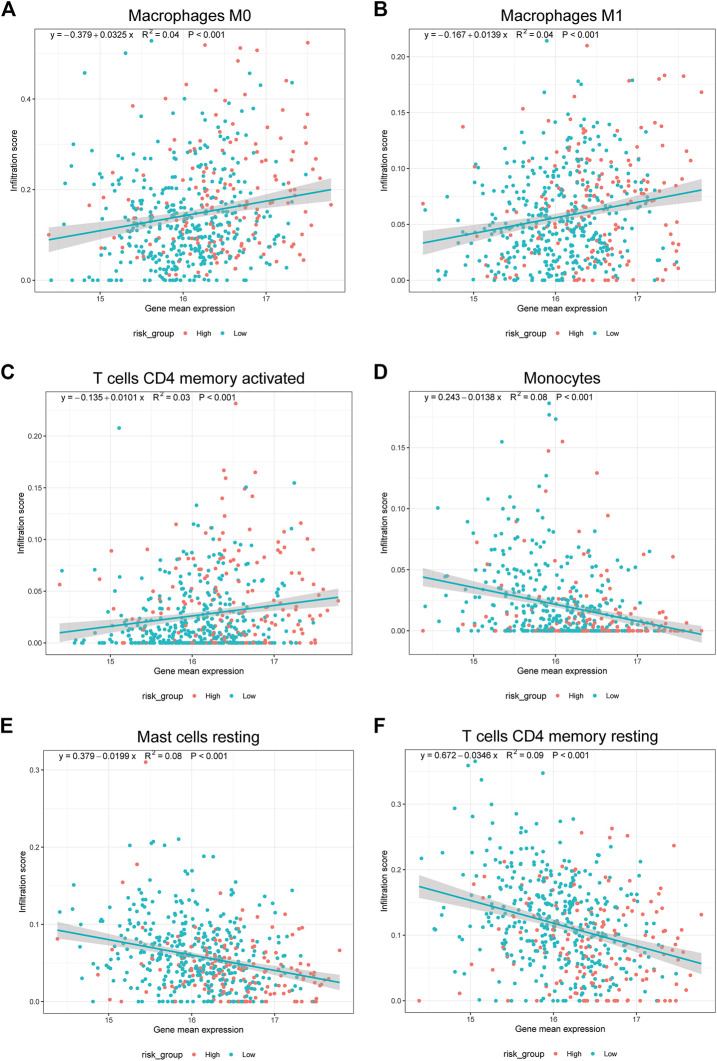
Prognostic model genes associated with immune cells. **(A–F)**. Correlation of mean gene expression in prognostic model with Macrophage M0, Macrophage M1, mast cell resting, Monocytes, T cells CD4 memory activated, T cells CD4 memory resting.

**FIGURE 9:**
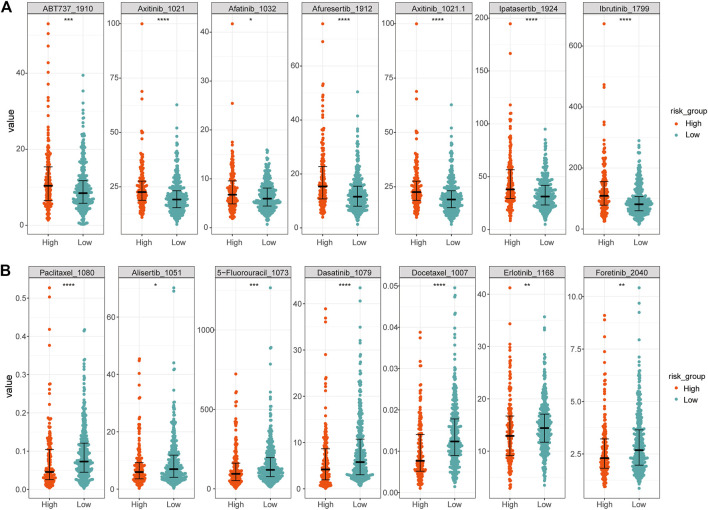
Differences in sensitivity of high and low risk groups to different drugs. **(A)** More sensitive drugs in the high-risk group. **(B)** Drugs with higher resistance in the high-risk group.

Finally, to reveal the relationship between the expression of 17 prognostic platinum resistance genes and the infiltration level of immune cells more directly, a scatter plot was drawn and the correlation curve was fitted by taking the expression values of 8 significant differential immune cells and prognostic platinum resistance genes in the CIBERSORT results. The results showed that the average expression value of prognostic platinum resistance genes was positively correlated with macrophage M0, macrophage M1 and T cells CD4 memory activated (Figures 8A–C), while mast cell resting, monocytes and T cells CD4 memory resting were negatively correlated (Figures 8D–F). Then, to further identify drugs that may interact with the high-risk group, we identified the drug sensitivity of TCGA-LUAD patients (Figure 9) according to the gene expression data of TCGA-LUAD patients and the GDSC database. The results showed that the high-risk group was sensitive to drugs ABT7371910, Axitinib1021, Afatinib1032, Afuresertib1912, Axitinib1021, Ipatasertib1924, and Ibrutinib1799, and had lower IC50 (Figure 9A). In contrast, the high-risk group was nonsensitive to Afatinib1032, Bortezomib1191, Dasatinib1079, Docetaxel1007, Erlotinib1168, Gefitinib1010, Lapatinib1558, ZM4474391050, Paclitaxel1080, and Tozasertib1096 (Figure 9B).

### 3.6 ceRNA network construction

**FIGURE 10:**
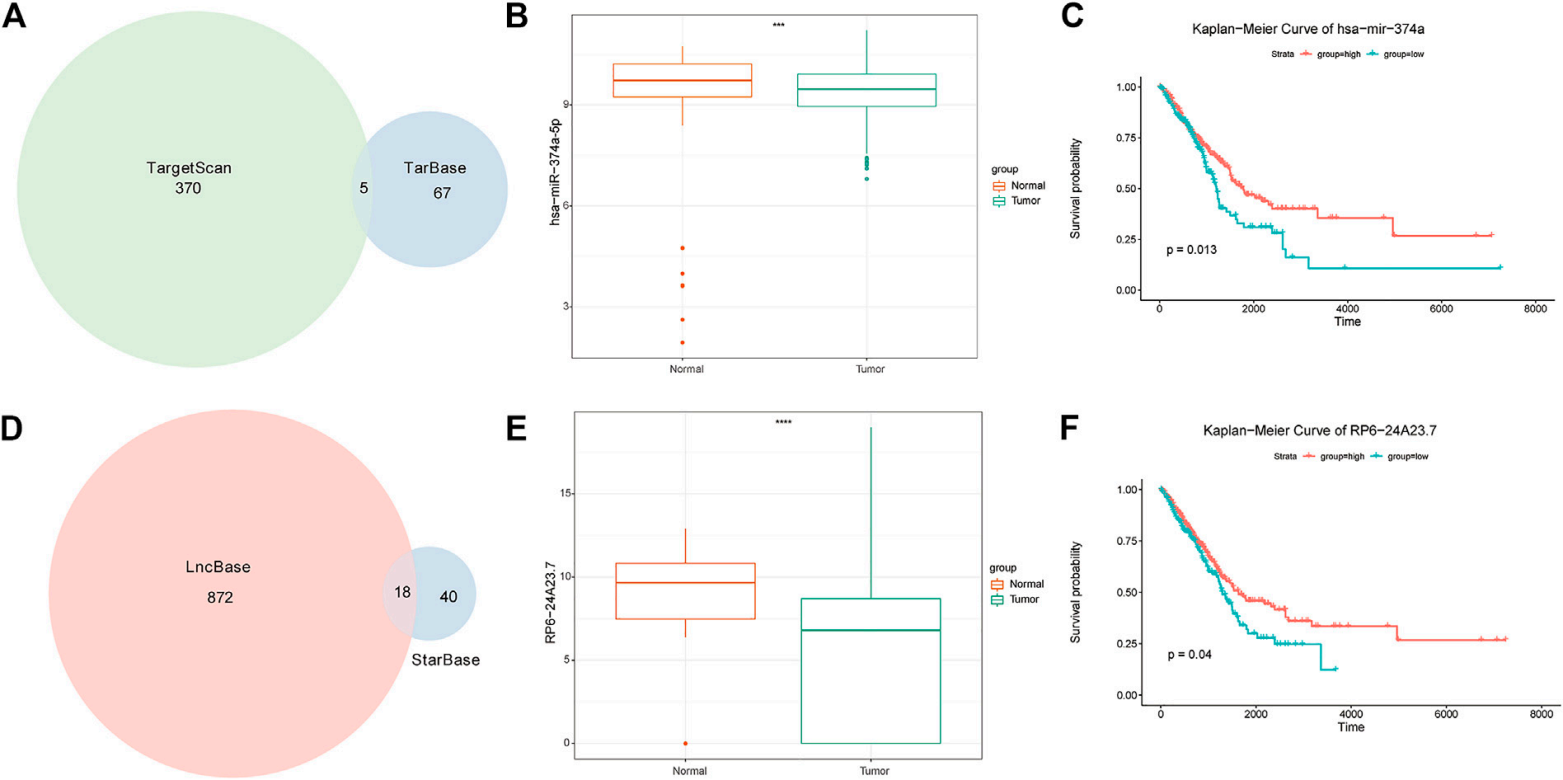
ceRNA analysis results. **(A)** Venn diagram of miRNA intersection of miRNAs in targerScan and tarbase databases. **(B)** mRNA expression difference between tumor group and normal group. **(C)** Survival analysis of miRNAs in high-risk and low expression groups. **(D)** Venn diagram of the interaction of miRNAs in lncBase predicted v2 and starBase V2. **(E)** LncRNA expression difference between tumor group and normal group. Survival analysis of **(F)** LncRNA in high and low expression groups.

In total, 17 PRR-DEGs related to prognosis were identified, 5 mi-RNA (Figure 10A) interacting with PRR-DEGs were identified based on Tarbase and Targetscan databases, and 18 lncRNA (Figure 10D) interacting with miRNA were found through lncBase predicted V.2 and the StarBase V2.0 databases, which constituted the ceRNA network. The difference in expression of lncRNA and miRNA between the acute tumor group and normal group (Figure 10B, C), as well as the KM survival of high- and low-risk groups, were analyzed. The results showed that among the five mi-RNA, only has-miR-374a-5p had different expression and survival curves between the tumor group and the normal group. The expression of HAS-MIR-374a-5P in the tumor group was lower, but its survival in the high-expression group was worse (Figure 10E). The expression of RP6-24A23.7 in lncRNA was also lower in the tumor group, and the prognosis was worse in the high expression group (Figure 10F).

## 4 Discussion

Although the incidence of lung cancer is lower than that of female breast cancer, the mortality rate remains the highest worldwide (Xia et al., 2022). In China, the lung cancer death rate is the highest among all cancers (Miller et al., 2016). Until 1995, landmark meta-analysis confirmed that cisplatin-based chemotherapy could significantly prolong the survival of NSCLC patients compared with meta-supportive treatment (Listed, 1995). Subsequent studies further confirmed the importance of chemotherapy in the treatment of NSCLC (Socinski et al., 2013). Since then, targeted therapies and immunotherapies have been developed. However, many patients cannot use targeted drugs because they are resistant to the drugs or because they contain driving genes; moreover, immunotherapy cannot be used because of unqualified immune indexes. As such, chemotherapy remains the best option for these patients, despite its toxic nature and strong side effects. Cisplatin-based chemotherapy is still the main method for the treatment of many cancers, but patients treated with platinum drugs will inevitably develop drug resistance.

In this study, we explored the mechanisms of drug resistance, prolonged drug resistance time, and longer survival time for patients. M6A is a methylation modification of RNA adenine (A), which is one of the most abundant modifications in eukaryotic mRNA. It is mainly regulated by the m6A methylation regulator (Chen et al., 2019; Liu et al., 2019). Previous studies have focused on the relationship between m6A and the occurrence and development of LUAD (Li et al., 2022; Ma et al., 2022; Qian et al., 2021), or the relationship between the m6A regulatory factor and chemotherapy resistance of small cell lung cancer (Zhang et al., 2021a; Zhang et al., 2021b). Some studies have also found that the m6A regulatory factor is closely related to LUAD resistance to erlotinib (Li K et al., 2021). As far as we know, our study is the first to explore the relationship between m6A and LUAD resistance to platinum. Some past studies have found that FTO promotes the growth of lung cancer cells (Li J et al., 2019), but in this study, the expression of FTO in the low-risk group was higher than that in the high-risk group, which suggests that the m6A regulatory gene may have changed in platinum-resistant patients with LUAD.

In this study, by comparing the genes of LUAD patients from the TCGA database with platinum resistance genes from A database of genes related to platinum resistance, 252 platinum resistance genes with significant differential expression were obtained, of which 161 were upregulated and 91 were downregulated. Among the significantly different drug resistance genes, LIN28B is the most upregulated gene. LIN28, a structurally conserved RNA-binding protein, is highly expressed in embryonic hepatocytes. It can promote rapid cell proliferation and is highly expressed in a variety of tumor tissues and tumor cell lines. The high expression of the LIN28 gene can increase the ability of liver cancer cells to metastasize to distant places (McDaniel et al., 2016), moreover, LIN28 can increase the resistance of ovarian and breast cancer cells to chemotherapy drugs by regulating let-7i (Yang et al., 2008). High expression of LIN28 can increase the insensitivity of lung cancer cells and pancreatic cancer cells to radiotherapy (Oh et al., 2010). However, when LIN28 is inhibited, the growth of NSCLC is reduced (Yang et al., 2019). Our research indicates that the high expression of LIN28B may lead to an increase in the resistance of LUAD cells to platinum drugs. This is helpful for further verification in subsequent experiments.

A PPI network diagram of upregulated and downregulated genes was constructed, and correlation analysis showed that the correlation between upregulated and downregulated genes was greater. The essence of oxidative stress is the imbalance of the oxidation–antioxidation system *in vivo*. However, the intracellular oxidation–reduction system of many tumor cells is out of balance, and so the drug resistance of LUAD cells is also closely related to oxidative stress (Winterbourn, 2008). There is DNA recombination in the GO pathway of gene enrichment, and the study shows that the occurrence and development of lung adenocarcinoma are closely related to it (Liang et al., 2022). We speculate that platinum resistance of lung adenocarcinoma is also closely related to it, and we will focus on it in the follow-up research. The research and development of lipopolysaccharide drugs have further improved drug utilization (Guo et al., 2019). In our down-regulated gene GO enrichment, top1 is the reaction pathway to lipopolysaccharide. We speculate that in patients with lung adenocarcinoma drug resistance, the metabolism of lipopolysaccharide substances decreases, which may lead to resistance to platinum drugs, which has a certain hint for us to improve platinum drugs. The top three GSEA enrichment sites were e2f targets, g2m checkpoint, and rgf beta signaling. E2f is a transcription factor gene family. Previous research found that E2F can regulate the expression of mitochondria-related genes, and the loss of this regulation leads to serious mitochondrial defects that affect cell metabolism and tumor cells (Benevolenskaya and Frolov, 2015). Yao et al. (Yao et al., 2020) found that E2F, the most abundant pathway in our GSEA analysis, is a potential biomarker and therapeutic target of colon cancer, which indicates that LUAD resistance to platinum is also closely related to the E2f family. The G2m checkpoint pathway is an important part of the cell cycle and is related to the occurrence and development of many tumors. A High G2M score is always related to the overall survival rate of pancreatic cancer (Oshi et al., 2020).

NMF, which can be used to solve the complex and excessive calculation issues caused by huge data, is a decomposition non-probability algorithm using matrix decomposition, belonging to the linear algebra algorithm group (Egger, 2022). NMF processes the data after TFIDF conversion by decomposing a matrix into two low-level matrices (Obadimu, 2019). We used the NMF algorithm for the molecular typing of PRR-DEGs and found that LUAD samples could be divided into two categories. We then used heatmap visualization to identify associations between PRR-DEGs and clinical information such as stage, race, age, and sex. Using PCA, we also found that the two categories of samples could be distinguished easily. In GSVA analysis, 4dk wnt beta-catenin signaling and hedgegcg signaling had the highest scores in C1. Wnt/β-catenin is a classic signal pathway, and the occurrence and development of many tumors are closely related to it, including colon cancer, hepatocellular carcinoma, desmoid tumor, pancreatic cancer, gastric cancer, melanoma, ovarian cancer, renal cancer (Guillen-Ahlers,2008), and prostate cancer (Robinson et al., 2008). Our analysis shows that the platinum resistance of LUAD is also related to Wnt/β. In contrast, g2 eckpoint and e2f scored higher in C2. Some studies (Li S et al., 2021) have shown that the expression of abnormal cyclin G2 is the key factor leading to the pathological process of cancer, including glioma. Among the platinum resistance genes in LUAD, the related gene of cyclin G2 is also very important and warrants attention.

To better guide clinical work, we constructed a prognostic model to evaluate the PRR-DEGs, and screened 252 PRR-DEGs using the LASSO regression method. There were 17 genes with a non-zero retention coefficient, and so we constructed a prognostic model with 17 genes; the influence coefficient of each gene was the coefficient of the LASSO regression results. Based on TCGA-LUAD data and a validation set (GSE26939), survival analysis was carried out to verify the prognostic efficacy of the prediction model, which confirmed that the prognosis of the high-risk group was poor. Moreover, risk factor analysis was used to evaluate the correlation trend between each gene and risk score. We found that genes such as HOXB7, NT5E, and KRT18 were positively correlated with risk score. Studies (Yan et al., 2022) have shown that the HOXB gene cluster contributes to cancer development; increased expression of HOXB3, HOXB6, HOXB7, HOXB8, and HOXB9 in LUAD patients is linked with poor overall survival (OS). Our data mining also illustrated the close relationship between the HOXB7 gene and LUAD platinum resistance. Past research (Dong et al., 2020) has also shown that NT5E levels are significantly higher in NSCLC tissues and cells. In our model, NT5E was also associated with the platinum resistance of LUAD, suggesting that the NT5E gene may be related to the development of lung cancer. The study has shown that the overexpression of ALDOA increases the migration and invasion of lung cancer cell lines *in vitro* and the formation of metastatic lung cancer *in vivo* (Chang et al., 2019). Our analysis suggests that ALDOA may also be associated with platinum drug resistance. Cystatin (CASPs) is an important regulator and executor of the apoptosis pathway. It has been found that the CASP family is closely related to the prognosis of non-small cell lung cancer (Lee et al., 2010). However, there is no study to explore the relationship between CASP and platinum resistance. Our analysis shows that CASP12, and CASP14 may be related to platinum resistance. This will guide the following research. The researchers have found that the deletion of FAT1 can accelerate the occurrence and malignant progression of tumors. In mouse and human squamous cell carcinomas, the loss of FAT1 function can promote tumorigenesis by inducing a mixed EMT state (Pastushenko et al., 2021). The other study has shown that FAT1 mutation is associated with better immunogenicity and ICI efficacy, which may be considered as a biomarker of patients who choose to receive immunotherapy (Zhang et al., 2022). The results of the expression of the same gene will be different under different treatment regimens. No one has studied the relationship between FAT1 and platinum resistance.FEN1 is the main component of the basic excision and repair pathway of the DNA repair system. Studies have shown that the high expression of the FEN1 gene is essential for the rapid proliferation of lung cancer cells, and the FEN1 gene can also increase the resistance of lung cancer cells to cisplatin (He et al., 2017). Studies have shown that the over-expression of GDF15 significantly inhibits the proliferation of NSCLC *in vitro* and *in vivo* (Lu et al., 2018). Through our analysis and prediction, GDF15 may be also related to platinum resistance of lung adenocarcinoma, which needs further verification by subsequent experiments. Studies have shown that high TXNRD1 protein levels are associated with shorter disease-free survival and postoperative distal metastasis-free survival in patients with NSCLC, including some individuals receiving platinum adjuvant chemotherapy (Delgobo et al., 2021; Guo et al., 2021), indicating that TXNRD1 is an important predictor of poor prognosis, which is consistent with our conclusion. Some studies have shown that UBE2S promotes the metastasis of lung adenocarcinoma cells by activating NF- κ B signal transduction, while other studies have shown that UBE2S regulates Wnt/β-catenin signal and promotes the progression of non-small cell lung cancer (Ho et al., 2021; Qin et al., 2020).In our predictive model, UBE2S is also an important factor in platinum resistance in patients with lung adenocarcinoma. The clinical prediction model established by Luo Yu et al. also shows that WFDC2 is an important factor affecting the prognosis of lung adenocarcinoma. Whether WFDC2 is also an important factor affecting platinum resistance in lung adenocarcinoma needs further experimental verification (Luo et al., 2022). Univariate Cox regression analysis showed that prognostic model genes, KRAS mutations, stages, and so on, were significantly correlated with OS. These univariate prognostic variables were used as covariates of multivariate Cox regression analysis. Except for CASP14, the other 16 prognostic model genes were independent prognostic factors related to OS. The nomogram model once again confirmed the reliability and prospective clinical applicability of the risk model. When the nomogram was used for calibration, the predicted results were highly correlated with the actual survival rate. This shows that our risk model has good clinical application value.

Focusing on the immune status of tumor patients will help us to explore the mechanisms of drug resistance and new therapeutic targets. Therefore, we used the CIBERSORT method to calculate the infiltration degree of immune cells in all samples; the results showed that resting T cells CD4 memory, resting dense cells, resting mast cell, and monocytes had higher infiltration degrees in the low-risk group than in the high-risk group. While activated T cells CD4 memory, macrophage M0, macrophage M1, and activated mast cells had higher infiltration degrees in the high-risk group compared with the low-risk group. From a scatterplot of the above eight different immune cells and platinum resistance genes, the average expression values of platinum resistance genes in prognosis were positively correlated with macrophage M0, macrophage M1 and activated T cells CD4 memory but negatively correlated with resting mast cell, monocytes, and resting T cells CD4 memory.

The TIDE score can be used to evaluate the potential clinical efficacy of immunotherapy in different immune-related gene prognostic model (IRGPI) subsets (Liu, 2018). The higher the TIDE prediction score, the higher the possibility of immune escape, suggesting that patients are less likely to benefit from immunotherapy. Compared with the high-risk group, the low-risk group had higher TIDE scores, indicating that people in the low-risk group were more likely to experience immune escape and could not benefit from immunotherapy. N6-methyladenosine, also called m6A, is a base modification widely existing in mRNA. The internal modification of mRNA can affect the RNA splicing, translation, stability, and epigenetics of some non-coding RNAs (Meyer and Jaffrey, 2017). By analyzing the differences in m6A regulatory factors between high- and low-risk groups, it was found that FTO, GPm6A, METTL3, and YTHDC2 expression was higher in the low-risk group. Among these, studies have shown that FTO plays the role of an oncogene in acute myeloid leukemia (Li et al., 2017) by regulating the level of m6A and promoting the occurrence and development of leukemia. Later, other studies showed that FTO plays a role as m6A demethylase in various life processes (Gokhale et al., 2016; Xiang et al., 2017). However, in this study, FTO was highly expressed in the low-risk group. We suggest that FTO is not closely related to platinum drug resistance in LUAD. The expressions of HNRNPA2B1, HNRNPC, TGF2BP1, IGF2BP2, IGF2BP3, and RBM15B were higher in the high-risk group, indicating that the above m6A regulatory factors play an important role in the mechanism of platinum resistance in LUAD. From drug sensitivity analysis, the high-risk group had lower IC50 to the drugs ABT7371910, Axitinib1021, Afatinib1032, Afuresertib1912, Axitinib1021, Ipatasertib1924, and Ibrutinib1799, indicating that the high-risk group is more sensitive to these drugs, but less sensitive to Afatinib1032, Bortezomib1191, Dasatinib1079, Docetaxel1007, Erlotinib1168, Gefitinib1010, Lapatinib1558, ZM4474391050, Paclitaxel1080, and Tozasertib1096. This has a certain clinical guiding significance for patients with LUAD after platinum resistance. Has-miR-374a-5p is related to the occurrence and development of pancreatitis (Wen et al., 2019), but the relationship between has-miR-374a-5p and platinum drug resistance of LUAD has not been explored. ceRNA analysis showed that has-miR-374a-5p is highly expressed in healthy individuals, and the higher the expression, the better the survival. RP6-24A23.7 is associated with lymphatic metastasis of LUAD (Yan et al., 2017). Morever, as we have demonstrated, may also be associated with platinum resistance in LUAD. The expression of RP6-24A23.7 was lower in the tumor group, and the survival was worse in the low expression group.These indicate that has-miR-374a-5p and RP6-24A23.7 were protective factors.

However, our research also had some limitations. First, in order to fully clarify the molecular mechanisms of resistance and development of platinum drugs in LUAD, microarray samples from platinum drugs in different degrees of LUAD are needed. Second, many biomarkers related to platinum resistance in LUAD still have no characteristics, and further bioinformatic analyses and experimental verifications are needed to clarify the biological function of these predictive genes in platinum resistance in LUAD. Unfortunately, because of the COVID-19 epidemic, our basic experimental process has been hindered. In future research, we will further use experiments to verify the mechanisms of drug resistance.

In summary, this study explored the characteristics of high- and low-risk groups by analyzing the biological process characteristics of platinum resistance genes in LUAD, established a prognosis model, and analyzed its m6A regulatory factors, immune infiltration, and drug sensitivity. Our results have significance for guiding clinical practice. We identified the potential targets and mechanisms of LUAD platinum resistance, laying the foundation for further research.

## Data Availability

Publicly available datasets were analyzed in this study. This data can be found here: TCGA (https://tcga-data.nci.nih.gov/tcga/), A database of genes related to platinum resistance (http://ptrc-ddr.cptac-data-view.org), GEO (https://www.ncbi.nlm.nih.gov/geo/query/acc.cgi?acc=GSE26939).
